# Orofacial Clefts and Maternal Risk Factors: A Population-Based Case–Control Study

**DOI:** 10.3390/children11070819

**Published:** 2024-07-04

**Authors:** Michele Santoro, Lorena Mezzasalma, Alessio Coi, Anna Pierini

**Affiliations:** 1 Unit of Epidemiology of Rare Diseases and Congenital Anomalies, Institute of Clinical Physiology, National Research Council, 56124 Pisa, Italy; 2 Foundation Gabriele Monasterio CNR-Regione Toscana, 56124 Pisa, Italy

**Keywords:** orofacial clefts, risk factors, case–control, cleft palate, cleft lip

## Abstract

Background/Objectives: Orofacial clefts (OFCs) are some of the most common congenital anomalies worldwide. The aim of this case–control study was to evaluate the association of OFCs with selected maternal characteristics. Methods: Data on isolated non-syndromic cases of OFCs were extracted from the population-based registry of congenital anomalies of Tuscany. A sample of live-born infants without any congenital anomaly was used as the control group. We investigated the association with sex and some maternal characteristics: age, body mass index, smoking, and education. Adjusted odds ratios (OR) were calculated using a logistic regression model. Analyses were performed for the total OFCs and separately for cleft lip (CL) and cleft palate (CP). Results: Data on 219 cases and 37,988 controls were analyzed. A higher proportion of males (57.9%) was observed, particularly for CL. A decreasing trend among the maternal age classes was observed (OR:0.81 (95%CI 0.70–0.94)). Underweight mothers had a higher prevalence of OFCs, in particular for CL (OR:1.88 (95%CI 1.08–3.26)). Conclusions: We found an association of OFCs with lower maternal age. The association with maternal age remains controversial and further epidemiological evidence is needed through multicenter studies. We observed that CL was more common in underweight mothers, suggesting actions of primary prevention.

## 1. Introduction

Orofacial clefts (OFCs) are a group of anomalies affecting the lips and the oral cavity. They are some of the most common congenital anomalies worldwide [1], with considerable ethnic and geographical variations. In Europe, according to the last updated data from EUROCAT, the European network of population-based registries for the epidemiological surveillance of congenital anomalies, the prevalence for this anomaly group in the period 2012–2021 was 14.6 per 10,000 births [2]. Despite their high prevalence, the etiology of OFCs remains largely unknown.

This congenital anomaly affects speech, hearing, feeding, aesthetics, and psychology, causing unfavorable health outcomes and negative impacts on social integration. Children born with OFCs have high morbidity, especially in the early years of life, representing a burden for the affected children and their families [1].

Some clefts are part of a syndrome but mainly occur as isolated anomalies (i.e., no other birth anomaly present) or in association with other major non-syndromic anomalies. Non-syndromic clefts are possibly caused by a combination of family history and genetics and exogenous determinants such as geographic factors and ethnic predisposition, socioeconomic conditions, improper diet, or exposure to teratogenic substances during pregnancy (alcohol, tobacco, or drugs) [3,4,5,6]. Even if the etiology of non-syndromic OFCs remains unknown, non-genetic factors play an important role in the development of these congenital anomalies [6].

A multidisciplinary approach is needed for the clinical management of children with OFCs [1] and much of the current research on OFCs concerns improving the quality of life (surgical and medical care, long-term rehabilitation of speech, hearing, cognition, and other long-lasting adverse outcomes). However, the primary prevention of some modifiable factors remains a major focus. Many studies have recognized the importance of family aggregation and, therefore, the relevance of the genetic component in the etiopathogenesis of this group of anomalies. Similarly, it is known that there may be an interaction between genetic components and environmental factors [7,8,9]. Identifying potential modifiable risk factors of OFCs represents an important source of information for developing educational programs and supporting prevention policies.

This study aimed to investigate the association between the occurrence of OFCs and some selected maternal characteristics using a population-based approach.

## 2. Materials and Methods

We conducted a population-based case–control study. We retrieved data on cases diagnosed from 1 January 2005 to 31 December 2017 from the population-based Registry of Congenital Defects of Tuscany (RTDC, from the Italian acronym “Registro Toscano dei Difetti Congeniti”). RTDC is a member of the EUROCAT network [10,11] and the International Clearinghouse for Birth Defects Surveillance and Research [12]. RTDC collects data on live births, fetal deaths with gestational age ≥ 20 weeks, and terminations of pregnancy for fetal anomaly following prenatal diagnosis. The collection of all cases diagnosed during the first year of life born to women living in Tuscany (Italy) is done through a large regional network that includes all obstetrical and maternity units, pediatric departments, pediatric cardiac surgery units, prenatal diagnostic centers, and medical genetics units. Tuscany is an Italian region with about 3,600,000 inhabitants and 22,000 births yearly.

Eligible for the current study were singleton cases with OFCs. Out of 311 cases extracted from RTDC, we excluded 23 cases with chromosomal anomalies, genetic syndromes, or Pierre Robin sequence. Furthermore, we also excluded 25 cases with established family history for OFCs—i.e., the presence of this anomaly was reported for the mother or father and some other members of their families. Among the remaining 263 cases, there were 219 isolated cases and 44 multiple cases, that is, OFCs co-occurring with one or more other structural anomalies. Among the 44 multiple cases of OFCs, 47.7% had a congenital heart defect, 18.2% an anomaly of the urinary system, 18.2% an anomaly of the limbs, 18.2% an anomaly of the urinary system, 9.1% an anomaly of the eye, and 20.5% other structural anomalies. After the exclusion of multiple cases, overall, 219 isolated cases of OFCs were finally included in the study.

A random sample of 10% of children born to mothers residing in Tuscany without any congenital anomaly was used as a control. Data were retrieved from birth certificates in the period 2005–2017 stratified by birth year.

The following maternal characteristics were considered: age, body mass index (BMI), education, and smoking. Maternal age at delivery/termination was selected in the 16–44 years range and categorized into four classes: 16–24, 25–29, 30–34, and 35–44 years. Plausible BMI values were considered in the range of 15–50, following the EUROCAT guidelines [13]. Self-reported pre-pregnancy weight and height were used to calculate maternal BMI (kg/m^2^), categorized according to WHO classification in the following levels: underweight (BMI < 18.5), normal weight (BMI 18.5–24.9), overweight (BMI 25–29.9), and obesity (BMI ≥ 30). Maternal education was stratified into three classes: low (primary and lower secondary level), medium (upper secondary level), and high (tertiary level). Smoking during pregnancy was dichotomized into smokers and nonsmokers.

In this paper, we conventionally use the terms OFCs as a whole group, cleft palate without cleft lip (CP), cleft lip without cleft palate (CL), cleft lip with cleft palate (CL & CP), and cleft lip with or without cleft palate (CL ± CP).

### Statistical Analysis

All analyses were performed for OFCs as a whole group and separately for the four subgroups of OFCs: CP, CL, CL & CP, and CL ± CP. The sex ratio of males versus females (M/F) with a 95% confidence interval of probability (95%CI) was calculated. A logistic regression model estimated the association of OFCs and maternal characteristics. Crude and adjusted odds ratio (OR) with 95%CI were calculated. The trend across the maternal age classes was also assessed. Statistical analysis was conducted using Stata version 16.0 (StataCorp LP, College Station, TX, USA).

## 3. Results

A total of 219 cases of OFCs and 37,988 newborns without any congenital anomaly were analyzed (Table 1). Looking at the distribution of the cases, about 30% of the mothers were over 35 years, 25% had a low educational level, 9% declared to smoke during pregnancy, 20% were overweight and 6% obese before pregnancy, and 25% came from a foreign country. Among the 219 cases, 79 were cleft palate without cleft lip, 57 were cleft lip without cleft palate, and 83 had a diagnosis of cleft lip with cleft palate. Overall, 140 cases had a diagnosis of cleft lip with or without cleft palate.

A higher proportion of males (57.9%) with OFCs was observed, particularly for cases with a diagnosis of CL (73.7%). Instead, a higher proportion of females (58.2%), even if not significant, was observed among the subjects with only CP (Table 2).

A lower proportion of isolated OFCs was observed for cases with high maternal age compared to those with low maternal age (<24 years) (Table 3). Mothers with age higher than 35 years had a lower risk of occurrence compared to young mothers (aOR 35–39 years:0.47 (95%CI 0.27–0.82); aOR 40–44 years:0.42 (95%CI 0.20–0.88)). A decreasing linear trend among the age classes was also observed (aOR:0.81 (95%CI 0.70–0.94)). Underweight mothers had an increased risk of OFCs (aOR:1.60 (95%CI 1.00–2.53)). We did not observe an association with overweight and obese mothers.

In all the subgroups of OFCs, we observed a decreasing prevalence with increasing maternal age (Table 4). In particular, a significant trend was observed for the subgroup of CL ± CP (aOR:0.82 (95%CI 0.68–0.99)). Prevalence of CP from mothers aged over 40 years was significantly lower than in young mothers (aOR:0.21 (95%CI 0.04–0.99)) A higher prevalence for underweight mothers was observed for the cases with a diagnosis of CL, in particular for CL ± CP (aOR:1.88 (95%CI 1.08–3.26).

We did not observe associations with the other investigated maternal risk factors (i.e., education, smoking). The results of the analyses conducted on all cases (i.e., isolated and associated with other structural anomalies) did not differ from those observed on only isolated cases (Supplementary Materials).

## 4. Discussion

In this population-based case–control study on isolated non-syndromic OFCs, we found a higher proportion of males in CL and a higher proportion of females in CP, a higher proportion of cases among mothers with low maternal age, and an association with low maternal BMI in cleft lip cases.

The different sex proportion in CL and CP is highly reported in the literature [6,8,14,15,16,17,18,19]. The reason for this different distribution is still unknown, but it suggests a specific sex-related genetic involvement [16,20,21].

We found a decreasing prevalence trend with the increase of maternal age. A meta-analysis of 2012, reporting studies until 2010 on OFCs and parental age, found an association with the older age of mothers [22]. Some subsequent studies also found an association between low maternal age and OFCs as a whole group [4,18,23,24] and CL alone [19]. A higher prevalence of OFCs, especially for CL ± CP in young mothers, was observed in a EUROCAT study on maternal age and non-chromosomal congenital anomalies [25]. Thus, the literature on the association of OFCs with maternal age seems controversial with inconclusive results. In addition, according to recent systematic reviews, it is advisable to investigate more in detail the role of both high and low maternal age [6,26]. It has been hypothesized that a few specific congenital anomalies or general adverse pregnancy outcomes are associated indirectly with young maternal age; rather, this can be an effect of association with nonbiologic factors, such as a few lifestyle factors, which may be more frequent in younger mothers [25,27]. A possible explanation might be that young mothers are less likely to use folic acid supplementation before pregnancy [28]. The preventive effects of folic acid on OFCs were reported in some observational studies, but the evidence remains inconsistent and needs further investigation [29,30]. The effect of maternal age remains unclear for the low and high age classes, whereas the intermediate ages seem to play a protective role [6]. Multicenter studies using population-based data are recommended to provide more insight to the association of OFCs with parental age.

In our previous investigation [31], maternal underweight was associated with a significantly increased risk of OFCs. The results were consistent with the findings of some studies [32,33] but in contrast with others [34,35] which observed a positive association with maternal obesity. In addition, in a large international population-based study, the risk of OFCs was reported for both underweight and overweight mothers [36]. In the current study, we confirm the previous result, and in particular, we found that the excess of risk was significant in the CL ± CP subgroup but also noticeable in the other subgroups of clefts. However, statistical significance was not achieved, probably due to the small number of cases. An inadequate diet in the periconceptional period with a low supply of nutrients necessary for proper embryonic development could partly explain the increased risk of OFCs in underweight mothers [32,37].

We did not observe in our study population an association with maternal smoking, even if this risk factor is consistently reported in the literature [1]. No association was found with maternal education which can be considered a proxy of socioeconomic status. Socioeconomic deprivation may have an impact on unhealthy lifestyles and evidence of the association of CL with lower socioeconomic status was reported by Lupo et al. [38].

Interesting information arose from the subgroup analysis. A recent study [19] has demonstrated the importance of analyzing isolated separately from multiple (i.e., with co-occurring anomalies) OFCs and evaluating the various risk factors in the different subgroups of clefts. The associations with the main risk factors found in our study (sex of the newborn, maternal age, and underweight) are not constantly detectable in all subgroups. Whether this dissimilarity is mainly due to a different etiopathogenesis of the single defect or to independent factors (e.g., the different number of cases in each subgroup) is unclear and will require further investigation.

### 4.1. Strengths

The population-based setting of this study represents an important strength of the study. We included all cases born to mothers residing in an area and not only from specific tertiary clinical centers, thus avoiding a selection bias. Furthermore, we examined OFCs as a whole group and in four distinct subgroups to evaluate the specific role attributable to some selected risk factors and to address potential differences among subgroups. Another strength is that analyses were performed only on isolated cases, excluding syndromic, multiple, and familial cases. Thus, the estimates of the association with the investigated risk factors can be considered more accurate.

### 4.2. Limitations

The study also has a few limitations. The first limitation is the low size of the study population, which generates a lower statistical precision of the estimates and does not allow us to explore in detail a few factors, such as the association with maternal ethnicity. Another limitation was the lack of information on maternal gestational and pre-gestational diabetes, whose association with OFCs is reported in the literature. However, the association with pre-gestational diabetes was more common when OFCs were associated with other structural anomalies [19,39], and the inclusion of only isolated cases in our analyses has limited this source of potential bias. Another limitation was that BMI was calculated using self-reported height and pre-pregnancy weight which may contribute to recall bias. Finally, other potential risk factors were not investigated due to the lack of data, and their effect on the estimates of association can not be excluded.

## 5. Conclusions

In this population-based case–control study, we found an association of OFCs with lower maternal age. The association of OFCs with maternal age remains controversial across different studies, and further epidemiological evidence is needed through multicenter studies. The role of young maternal age can depend on biological and not biological factors, and more multidisciplinary investigation is suggested. We found that cases of CL were more common in underweight mothers, suggesting actions of primary prevention policy. OFCs are among the most common congenital anomalies with a high burden on individuals and their families. Investigating the role of non-genetic risk factors is a fundamental contribution to preventing these anomalies and multicenter studies using a multi-database approach are recommended.

## Figures and Tables

**Table 1:** Characteristics of mothers of cases with isolated orofacial clefts and controls, years 2005–2017.

	Controls	All OFCs ^§^
	N = 37,988	N = 219
Maternal characteristics	n (%)	n (%)
**Age** (years)		
16–24	3371 (8.9)	27 (12.4)
25–29	7569 (19.9)	48 (22.1)
30–34	12,912 (34.0)	78 (36.0)
35–39	10,808 (28.5)	50 (23.0)
40–44	3308 (8.7)	14 (6.5)
missing	20	2
**Education**		
High	10,364 (28.0)	51 (25.5)
Medium	16,282 (44.0)	98 (49.0)
Low	10,330 (28.0)	51 (25.5)
Missing	1012	19
**Body mass index** (kg/m^2^)		
<18.5 (underweight)	3024 (8.2)	22 (11.6)
18.5–24 (normal weight)	24,735 (67.0)	119 (63.0)
25–29 (overweight)	6652 (18.0)	37 (19.6)
≥30 (obesity)	2507 (6.8)	11 (5.8)
missing	1070	30
**Smoking**		
Nonsmoker	30,014 (91.1)	183 (90.6)
Smoker	2928 (8.9)	19 (9.4)
Missing	5046	17

Notes: ^§^ All cases with isolated OFCs (orofacial clefts).

**Table 2:** Proportion of males and sex ratio (male/female) with 95% confidence interval of isolated cases of orofacial clefts by type, years 2005–2017.

	% Male	Sex Ratio (M/F)	95%CI
Orofacial clefts	57.9	1.37	1.05–1.80
Cleft palate	41.8	0.72	0.46–1.12
Cleft lip	73.7	2.80	1.55–5.05
Cleft lip with cleft palate	62.5	1.67	1.06–2.62
Cleft lip with or without cleft palate	67.2	2.04	1.43–2.92

(M/F): (male/female); 95%CI: 95% confidence interval.

**Table 3:** Number of cases, crude and adjusted odds ratio with 95% confidence interval of isolated orofacial clefts by maternal characteristic, years 2005–2017.

Maternal Risk Factors	N	OR	95%CI	aOR *	95%CI
**Age (years)**					
16–24 (ref.)	27	1		1	
25–29	48	0.79	0.49–1.27	0.67	0.39–1.13
30–34	78	0.75	0.49–1.17	0.62	0.37–1.04
35–39	50	0.58	0.36–0.92	0.47	0.27–0.82
40–44	14	0.53	0.28–1.01	0.42	0.20–0.88
*Trend*		*0.85*	*0.75–0.96*	*0.81*	*0.70–0.94*
**Education**					
High (ref.)	51	1		1	
Medium	98	1.22	0.87–1.71	0.99	0.69–1.43
Low	51	1.00	0.68–1.48	0.82	0.53–1.28
**BMI (kg/m^2^)**					
Normal (ref.)	119	1		1	
Underweight	22	1.51	0.96–2.39	1.60	1.00–2.53
Overweight	37	1.16	0.80–1.67	1.28	0.88–1.86
Obesity	11	0.91	0.49–1.69	0.92	0.45–1.76
**Smoking**					
Nonsmoker (ref.)	183	1		1	
Smoker	19	1.06	0.66–1.71	1.06	0.64–1.76

N: number of cases; OR: odds ratio; 95%CI: 95% confidence interval; aOR: adjusted odds ratio; * adjusted by maternal characteristics and sex of the newborn.

**Table 4:** Adjusted odds ratio with 95% confidence interval of cleft palate without cleft lip, cleft lip without cleft palate, cleft lip with cleft palate, and cleft lip with or without cleft palate, by maternal characteristic, years 2005–2017.

Maternal Risk Factors	CP (n = 79)		CL (n = 57)		CL & CP (n = 83)		CL ± CP (n = 140)	
	aOR *	95%CI	aOR *	95%CI	aOR *	95%CI	aOR *	95%CI
**Age (years)**								
16–24 (ref.)	1		1		1		1	
25–29	0.59	0.25–1.42	1.03	0.35–2.97	0.54	0.23–1.30	0.71	0.36–1.39
30–34	0.60	0.26–1.37	0.79	0.27–2.29	0.55	0.24–1.26	0.64	0.33–1.23
35–39	0.54	0.22–1.29	0.70	0.23–2.15	0.30	0.12–0.78	0.44	0.21–0.89
40–44	0.21	0.04–0.99	0.57	0.13–2.54	0.50	0.17–1.50	0.54	0.22–1.30
*Trend*	*0.80*	*0.63–1.02*	*0.86*	*0.65–1.14*	*0.79*	*0.62–1.01*	*0.82*	*0.68–0.99*
**Education**								
High (ref.)	1		1		1		1	
Medium	1.47	0.77–2.83	1.04	0.51–2.14	0.68	0.38–1.21	0.81	0.52–1.26
Low	1.22	0.56–2.63	1.01	0.44–2.33	0.50	0.24–1.03	0.67	0.39–1.16
**Body mass index** (kg/m^2^)								
Normal (ref.)	1		1		1		1	
Underweight	1.14	0.48–2.69	1.88	0.82–4.31	1.88	0.90–3.92	1.88	1.08–3.26
Overweight	1.21	0.66–2.23	1.14	0.54–2.42	1.47	0.79–2.72	1.32	0.82–2.13
Obesity	0.45	0.11–1.88	0.99	0.30–3.27	1.43	0.56–3.66	1.23	0.59–2.58
**Smoking**								
Nonsmoker (ref.)	1		1		1		1	
Smoker	0.82	0.33–2.06	1.37	0.57–3.27	1.07	0.46–2.51	1.20	0.65–2.21

CP: cleft palate without cleft lip; CL: cleft lip without cleft palate; CL & CP: cleft lip with cleft palate; CL ± CP: cleft lip with or without cleft palate; aOR: adjusted odds ratio; 95%CI: 95% confidence interval; * adjusted by maternal characteristics and sex of the newborn.

## Data Availability

The datasets generated and/or analyzed during the current study are not publicly available due to National and EU data protection laws. The data analyzed in this study were subject to the following licenses/restrictions: the data owner is the Tuscany Region. Data are however available from the authors upon reasonable request and with permission of the Tuscany Region.

## Supplementary Materials

The following supporting information can be downloaded at: https://www.mdpi.com/article/10.3390/children11070819/s1, Table S1. Odds Ratio of orofacial clefts (isolated and multiple) by maternal characteristic, years 2005–2017
