# Supplementary material for: Orofacial Clefts and Maternal Risk Factors: A Population-Based Case–Control Study
Source: Children (Basel). 2024 Jul 4;11(7):819. doi: 10.3390/children11070819 (PMC11274858; doi:10.3390/children11070819)
Supplement: Supplementary file 1 [file children-11-00819-s001.zip › children-3039986-supplementary.pdf]

Supplementary Table S1

OROFACIAL CLEFTS (ISOLATED AND MULTIPLE)

Crude Odds Ratio (OR) and adjusted Odds Ratio (aOR) with 95% confidence interval (CI) of orofacial clefts (isolated and multiple), by maternal characteristic, years 2005-2017

Orofacial clefts (n=263)

	**OR**	**95%CI**	**aOR***	**95%CI**
**Maternal Age**				
16-24 (ref.)	1		1	
25-29	0.84	0.54-1.28	0.72	0.44-1.17
30-34	0.73	0.49-1.09	0.57	0.35-0.93
35-39	0.61	0.40-0.94	0.44	0.26-0.73
40-44	0.54	0.30-0.98	0.42	0.21-0.85
trend	*0.86*	*0.77-0.96*	*0.79*	*0.68-0.91*
**Maternal Education**				
high (ref.)	1		1	
medium	1.24	0.91-1.70	1.01	0.71-1.43
low	1.00	0.70-1.44	0.83	0.55-1.26
**Maternal BMI**				
normal (ref.)	1		1	
underweight	1.58	1.03-2.40	1.65	1.08-2.53
overweight	1.07	0.75-1.53	1.18	0.82-1.70
obesity	1.02	0.59-1.78	1.04	0.59-1.86
**Smoking**				
no smoker (ref.)	1		1	
smoker	0.94	0.59-1.49	0.91	0.55-1.50

*Adjusted by maternal characteristics and sex of the newborn.

Cleft palate (n=100)

	OR	95%CI	aOR*	95%CI
**Maternal Age**				
16-24 (ref.)	1		1	
25-29	0.89	0.46-1.74	0.76	0.37-1.55
30-34	0.67	0.35-1.26	0.49	0.23-1.02
35-39	0.60	0.31-1.17	0.45	0.21-0.99
40-44	0.24	0.07-0.83	0.23	0.06-0.86
trend	*0.78*	*0.66-0.94*	*0.74*	*0.59-0.92*
**Maternal Education**				
high (ref.)	1		1	
medium	1.83	1.06-3.19	1.45	0.80-2.60
low	1.42	0.76-2.64	1.15	0.58-2.29
**Maternal BMI**				
normal (ref.)	1		1	
underweight	1.49	0.76-2.92	1.54	0.78-3.04
overweight	1.01	0.57-1.80	1.08	0.60-1.92
obesity	0.90	0.36-2.24	0.93	0.37-2.35
**Smoking**				
no smoker (ref.)	1		1	
smoker	0.70	0.31-1.60	0.62	0.25-1.54

*Adjusted by maternal characteristics and sex of the newborn.

Cleft lip (n=66)

	OR	95%CI	aOR*	95%CI
**Maternal Age**				
16-24 (ref.)	1		1	
25-29	1.51	0.56-4.11	1.03	0.36-2.98
30-34	1.20	0.46-3.16	0.95	0.33-2.70
35-39	1.06	0.39-2.88	0.78	0.26-2.34
40-44	0.61	0.15-2.56	0.58	0.13-2.59
trend	*0.89*	*0.71-1.11*	*0.88*	*0.67-1.15*
**Maternal Education**				
high (ref.)	1		1	
medium	1.23	0.66-2.30	1.07	0.54-2.13
low	1.00	0.49-2.05	1.04	0.46-2.32
**Maternal BMI**				
normal (ref.)	1		1	
underweight	1.64	0.73-3.69	1.73	0.76-3.93
overweight	1.06	0.53-2.15	1.15	0.56-2.34
obesity	1.13	0.40-3.18	0.90	0.27-2.95
**Smoking**				
no smoker (ref.)	1		1	
smoker	1.12	0.48-2.60	1.27	0.54-3.02

*Adjusted by maternal characteristics and sex of the newborn.

Cleft lip with Cleft palate (n=97)

	OR	95%CI	aOR*	95%CI
**Maternal Age**				
16-24 (ref.)	1		1	
25-29	0.54	0.27-1.10	0.53	0.23-1.21
30-34	0.62	0.33-1.15	0.49	0.22-1.10
35-39	0.47	0.24-0.92	0.27	0.11-0.68
40-44	0.80	0.36-1.77	0.52	0.18-1.47
trend	*0.92*	*0.76-1.10*	*0.79*	*0.62-1.00*
**Maternal Education**				
high (ref.)	1		1	
medium	0.89	0.54-1.44	0.67	0.38-1.18
low	0.75	0.43-1.33	0.50	0.25-1.02
**Maternal BMI**				
normal (ref.)	1		1	
underweight	1.64	0.80-3.35	1.73	0.84-3.58
overweight	1.16	0.63-2.11	1.36	0.74-2.50
obesity	1.10	0.44-2.77	1.32	0.52-3.37
**Smoking**				
no smoker (ref.)	1		1	
smoker	1.09	0.53-2.27	1.02	0.43-2.38

*Adjusted by maternal characteristics and sex of the newborn.

Cleft lip with or without Cleft palate (n=163)

	OR	95%CI	aOR*	95%CI
**Maternal Age**				
16-24 (ref.)	1		1	
25-29	0.80	0.45-1.40	0.69	0.36-1.33
30-34	0.77	0.46-1.30	0.64	0.34-1.21
35-39	0.62	0.36-1.08	0.43	0.21-0.86
40-44	0.75	0.38-1.50	0.56	0.24-1.31
trend	*0.90*	*0.79-1.04*	*0.82*	*0.69-0.99*
**Maternal Education**				
high (ref.)	1		1	
medium	1.01	0.69-1.48	0.81	0.53-1.26
low	0.84	0.54-1.31	0.69	0.40-1.17
**Maternal BMI**				
normal (ref.)	1		1	
underweight	1.64	0.96-2.80	1.73	1.00-2.98
overweight	1.12	0.71-1.76	1.26	0.79-2.01
obesity	1.11	0.56-2.21	1.12	0.54-2.35
**Smoking**				
no smoker (ref.)	1		1	
smoker	1.10	0.64-1.92	1.13	0.62-2.07

*Adjusted by maternal characteristics and sex of the newborn.
